# Hierarchical cross-modal spatiotemporal attention network for crop yield prediction in complex Himalayan agroecosystems

**DOI:** 10.3389/fpls.2026.1933033

**Published:** 2026-10-14

**Authors:** Shabir Ahmad Mir, Umarfarooque Momin, Sudeep Kumar E., Itfaq Ahmad Mir, Ab Rouf Khan, G. M. Mir

**Affiliations:** 1 Agricultural Research Information System (ARIS), SKUAST of Kashmir, Srinagar, Jammu and Kashmir, India; 2 University of Agricultural Sciences, Dharwad, Karnataka, India; 3 Faculty of Veterinary Sciences and Animal Husbandry, SKUAST of Kashmir, Shuhama Alusteng, Ganderbal, India; 4 Centre for Artificial Intelligence and Machine Learning, SKUAST of Kashmir, Srinagar, Kashmir, India

**Keywords:** Crop yield prediction, Multimodal data fusion, Himalayan apple orchards, Spatio-temporal attention, Soil memory, Uncertainty quantification, Precision agriculture, Climate-smart agriculture

## Abstract

**Introduction:**

Accurate crop yield prediction is essential for precision agriculture, climate-resilient farm management, and timely decision-making. However, yield prediction remains challenging because of complex, nonlinear, and multi-scale interactions among crop physiology, soil conditions, weather variability, and management practices. Conventional statistical, machine learning, and deep learning approaches, including convolutional neural networks (CNNs), recurrent neural networks (RNNs), and long short-term memory (LSTM) networks, often have limitations in capturing long-range temporal dependencies, hierarchical spatial relationships, heterogeneous data modalities, and predictive uncertainty.

**Methods:**

This study proposes a Hierarchical Cross-Modal Spatio-Temporal Attention Network (HCM-STAN) for crop yield prediction using a unified attention-based framework for integrating heterogeneous agro-environmental data. The architecture incorporates four principal components: (i) hierarchical spatio-temporal attention operating across tree, block, orchard, and regional scales; (ii) a cross-modal attention module for adaptive fusion of IoT-based soil sensor observations, weather data, plant physiological measurements, and drone-based multispectral imagery; (iii) a soil-aware attention head that explicitly models soil memory, hysteresis effects, and root-zone dynamics; and (iv) an uncertainty-aware prediction module combining Monte Carlo dropout and quantile regression to generate calibrated prediction intervals. The framework was evaluated using a multi-source dataset collected from Himalayan apple orchards during 2023–2026, comprising 1,247 monitored trees across three commercial orchards.

**Results:**

The proposed HCM-STAN achieved an R^2^ of 0.89 for four-week-ahead crop yield prediction, representing a 15.6% improvement over the CNN-LSTM baseline. Furthermore, zero-shot transfer to mango orchards achieved an R^2^ of 0.67, corresponding to approximately 75% of the performance obtained under full training.

**Discussion:**

The results demonstrate that integrating hierarchical spatio-temporal representation learning, cross-modal attention, soil-aware modelling, and uncertainty estimation can effectively address the heterogeneous and dynamic nature of crop yield prediction in complex mountain agroecosystems. The observed zero-shot transfer performance further indicates the potential of HCM-STAN to generalize across crop systems without crop-specific retraining. Overall, the proposed framework provides a promising foundation for reliable, uncertainty-aware yield forecasting and can support precision agriculture, climate-smart farm management, and data-driven decision-making in heterogeneous agroecosystems.

## 1 Introduction

Crop yield prediction is a critical component of precision agriculture, enabling farmers to optimize resource allocation, anticipate market conditions, and mitigate climate-related risks (van Klompenburg et al., 2020; Mir et al., 2008). However, accurate prediction remains challenging due to the complex, nonlinear, and multi-scale nature of agro-ecological systems (Chlingaryan et al., 2018). Traditional approaches, ranging from statistical regression to classical machine learning, have demonstrated inherent limitations in capturing the spatio-temporal dependencies that characterize crop growth dynamics (Liakos et al., 2018).

Recent advances in deep learning, particularly recurrent neural networks (RNNs) and long short-term memory (LSTM) networks, have improved yield prediction by modeling temporal sequences (Khaki and Wang, 2019). Convolutional neural networks (CNNs) have been employed to extract spatial features from satellite and drone imagery (You et al., 2017). Hybrid CNN-LSTM architectures have achieved state-of-the-art results by combining spatial feature extraction with temporal sequence modeling (Sharma et al., 2022).

Despite these advances, existing approaches face three fundamental limitations. First, they struggle with long-range temporal dependencies. The vanishing gradient problem in RNNs and LSTMs limits their ability to relate early-season conditions (e.g., soil moisture during planting) to late-season outcomes (e.g., fruit quality at harvest) (Hochreiter and Schmidhuber, 1997). Second, most models treat multi-modal data sources independently, missing the crucial cross-modal interactions that drive crop development (e.g., how soil moisture affects canopy temperature, which influences pest pressure) (Kamilaris and Prenafeta-Boldú, 2018). Third, the majority of existing models operate as black boxes, providing predictions without uncertainty estimates or causal explanations, limiting their utility for decision-making (Wolfert et al., 2017).

Attention mechanisms, originally developed for natural language processing (Vaswani et al., 2017), offer a paradigm shift for agricultural time series modeling. Unlike sequential models, attention computes direct pairwise relationships between all time steps, enabling the capture of long-range dependencies without information decay. When extended to spatial dimensions, attention can model interactions across field locations, learning, for example, how pest pressure propagates from one area to another (Lal et al., 2026).

This paper presents a comprehensive technical framework for spatio-temporal attention applied to crop yield prediction, with four key contributions:

1. A hierarchical spatio-temporal attention architecture that operates at multiple scales (individual tree, block, orchard, regional), capturing multi-resolution agro-ecological dynamics.
2. A cross-modal attention mechanism that fuses heterogeneous data streams including IoT soil sensors, weather stations, plant physiology monitors, and drone multispectral imagery.
3. A soil-aware attention head that explicitly models soil memory, hysteresis, and root-zone coupling, addressing a critical gap in existing deep learning approaches.
4. Uncertainty quantification via Monte Carlo dropout and quantile regression, providing calibrated confidence intervals for risk-aware decision support.

The framework is validated on a comprehensive dataset from Himalayan apple orchards (2023–2026), comprising 1,247 monitored trees across three orchards with hourly to weekly sampling frequencies. Experimental results demonstrate that the proposed framework achieves R^2^ = 0.89 for yield prediction at 4-week horizons, outperforming CNN-LSTM baselines by 15.6%. Zero-shot transfer to mango orchards achieves R^2^ = 0.67, representing 75% of full-training performance.

The remainder of this paper is organized as follows. Section 2 reviews related work in crop yield prediction and attention mechanisms. Section 3 presents the problem formulation. Section 4 describes the proposed technical framework in detail. Section 5 reports experimental results and ablation studies. Section 6 discusses practical deployment considerations. Section 7 concludes with limitations and future directions.

## 2 Related work

### 2.1 Crop yield prediction methods

Crop yield prediction methods have evolved through three distinct generations. First-generation methods employed statistical approaches including linear regression, ARIMA, and crop simulation models such as DSSAT and APSIM (Jones et al., 2003; Keating et al., 2003; Mir et al., 2008). While simulation models encode mechanistic understanding of plant physiology, they require extensive calibration and are computationally intensive for spatial scaling (van Ittersum et al., 2003).

Second-generation methods leveraged machine learning algorithms including Random Forest, Support Vector Machines, and Gradient Boosting Machines (Jeong et al., 2016; Gonzalez-Sanchez et al., 2014). These methods demonstrated improved accuracy by capturing nonlinear relationships but typically aggregate temporal data into fixed windows (e.g., monthly averages), losing critical phenological information (Cai et al., 2019).

Third-generation methods employ deep learning architectures. CNNs have been widely used for spatial feature extraction from satellite imagery (You et al., 2017; Cho et al., 2014; Wang et al., 2018). RNNs and LSTMs have been applied to temporal sequences of weather and soil data (Khaki and Wang, 2019; Haider et al., 2019). Hybrid CNN-LSTM architectures have become the state of the art, achieving R^2^ values of 0.70–0.85 across various crops and regions (Sharma et al., 2022, Sharma et al., 2022; Zhang et al., 2023).

Despite these advances, CNN-LSTM models remain limited in their ability to capture very long-range dependencies. For apple orchards, early-season soil moisture conditions during bud break (March–April) can significantly affect fruit set in June and final yield in September, representing a temporal gap of 90–120 days. Standard LSTMs with 256 hidden units have an effective memory of approximately 30–50 days, insufficient for capturing these dependencies (Hochreiter and Schmidhuber, 1997).

### 2.2 Attention mechanisms in agriculture

Attention mechanisms have recently been introduced to agricultural applications. The Transformer architecture, which relies entirely on attention, has been applied to crop yield prediction with promising results (Lal et al., 2026; Rivas et al., 2022). Unlike LSTMs, Transformers compute direct pairwise attention between all time steps, enabling the capture of arbitrarily long-range dependencies.

Spatio-temporal attention extends the Transformer to model both spatial and temporal dimensions simultaneously. This is particularly relevant for agriculture because field conditions are spatially autocorrelated (nearby locations are more similar than distant locations) and temporally autocorrelated (conditions persist across time). Several studies have demonstrated that spatio-temporal attention outperforms CNN-LSTM for tasks including soil moisture prediction (Zhang et al., 2024), pest outbreak forecasting (Islam et al., 2023), and crop yield estimation (Srilatha and Sirisati, 2026).

### 2.3 Soil modeling in deep learning

A critical gap in existing deep learning approaches for agriculture is the treatment of soil. Most models treat soil moisture as an exogenous input variable rather than a dynamical system with memory and hysteresis (Li et al., 2024). However, soil physics literature has long established that soil moisture exhibits multi-week memory and that the relationship between soil water content and plant available water is hysteretic (Richards, 1931; Vereecken et al., 2008).

Recent work has attempted to incorporate soil memory using recurrent architectures (Fang et al., 2021). However, these approaches typically do not model depth-dependent dynamics or root-zone coupling. Our framework addresses this gap with a specialized soil-aware attention head that explicitly models soil memory via gated recurrent attention, hysteresis via direction-aware attention, and root-zone coupling via depth cross-attention.

### 2.4 Uncertainty quantification in yield prediction

Most crop yield prediction models provide point estimates without uncertainty quantification. This limits their utility for risk-averse decision-making. Bayesian deep learning methods, including Monte Carlo dropout (Gal and Ghahramani, 2016) and variational inference (Kingma and Welling, 2014), have been applied to agricultural time series with promising results (Zhang et al., 2023). Quantile regression provides an alternative approach that does not assume a parametric distribution (Koenker and Hallock, 2001). Our framework combines both approaches to provide well-calibrated prediction intervals.

## 3 Problem formulation

### 3.1 Input-output specification

Let us define the crop yield prediction problem as follows. Consider a set of spatial locations L (sensor nodes, trees, or grid cells) monitored over a temporal sequence of T time steps covering a growing season. At each time step t ∈ {1, …, T} and location l ∈ L, we observe a multi-modal feature vector:


$${\text{x}}_{1}\left(\text{t}\right)=\left[{\text{s}}_{1}\left(\text{t}\right),{\text{w}}_{1}\left(\text{t}\right),{\text{p}}_{1}\left(\text{t}\right),{\text{d}}_{1}\left(\text{t}\right)\right],\text{where:}$$

$$\begin{array}{l} \text{Soil Modality [}s_{l}\left(t\right)\in R^{D}s\text{] is Soil moisture (3 depths), temperature (3 depths), NPK, salinity, and microbial activity;}\\ \text{Weather Modality [}w_{l}\left(t\right)\in R^{D}w\text{] is Air temperature, humidity, precipitation, solar radiation, and wind speed;}\\ \text{Physiology Modality [}p_{l}\left(t\right)\in R^{D}p\text{] is Sap flow, fruit diameter, and trunk tilt (dendrometer) and}\\ \text{Drone Modality [}d_{l}\left(t\right)\in R^{D}d\text{] is Multispectral (NDVI, NDRE), thermal (CWSI), hyperspectral features} \end{array}$$

Additionally, we observe time-invariant features including management practices (irrigation schedule, fertilization, pruning) and soil texture classification.

The prediction task: Given observed features from time steps 1 through t_current (current date, typically 4–6 weeks before harvest), predict final yield y ∈ R^+^ (kg/tree or tons/hectare) at harvest time T.

### 3.2 Spatio-temporal dependencies

Crop yield is governed by three types of dependencies that any predictive model must capture:

Temporal dependencies: Current yield is a function of the entire historical sequence of environmental conditions, with varying importance across phenological phases. For apples, conditions during flowering (April–May) are generally more predictive than conditions during vegetative growth (June–July).

Spatial dependencies: Conditions at nearby locations are correlated due to shared soil type, microclimate, and management. Pest pressure and disease spread exhibit spatial contagion.

Cross-modal dependencies: Different data modalities interact. For example, soil moisture affects canopy temperature (via evaporative cooling), which affects drone thermal imagery. Weather forecasts affect future soil moisture, which affects sap flow.

### 3.3 Technical requirements

Let f_θ_ be a neural network with parameters θ that maps input sequences X = {x_l_(t)} to yield prediction $\hat{y}$. The model should satisfy:

Temporal causality [fθ must not use future information to predict the present];Spatial locality bias [nearby locations should have stronger default interactions];Phenological alignment [model should be sensitive to timing relative to crop growth stages] Calibrated uncertainty [prediction intervals should have correct empirical coverage]; and Interpretability [model should provide explanations for its predictions].

## 4 Technical framework

### 4.1 System overview

The proposed framework consists of five sequential modules, as illustrated in **Figure 1**. System architecture diagram showing data flow from raw sensors through tokenization, hierarchical spatio-temporal attention, cross-modal fusion, soil-aware attention, and uncertainty quantification to yield prediction.

**Figure 1:**
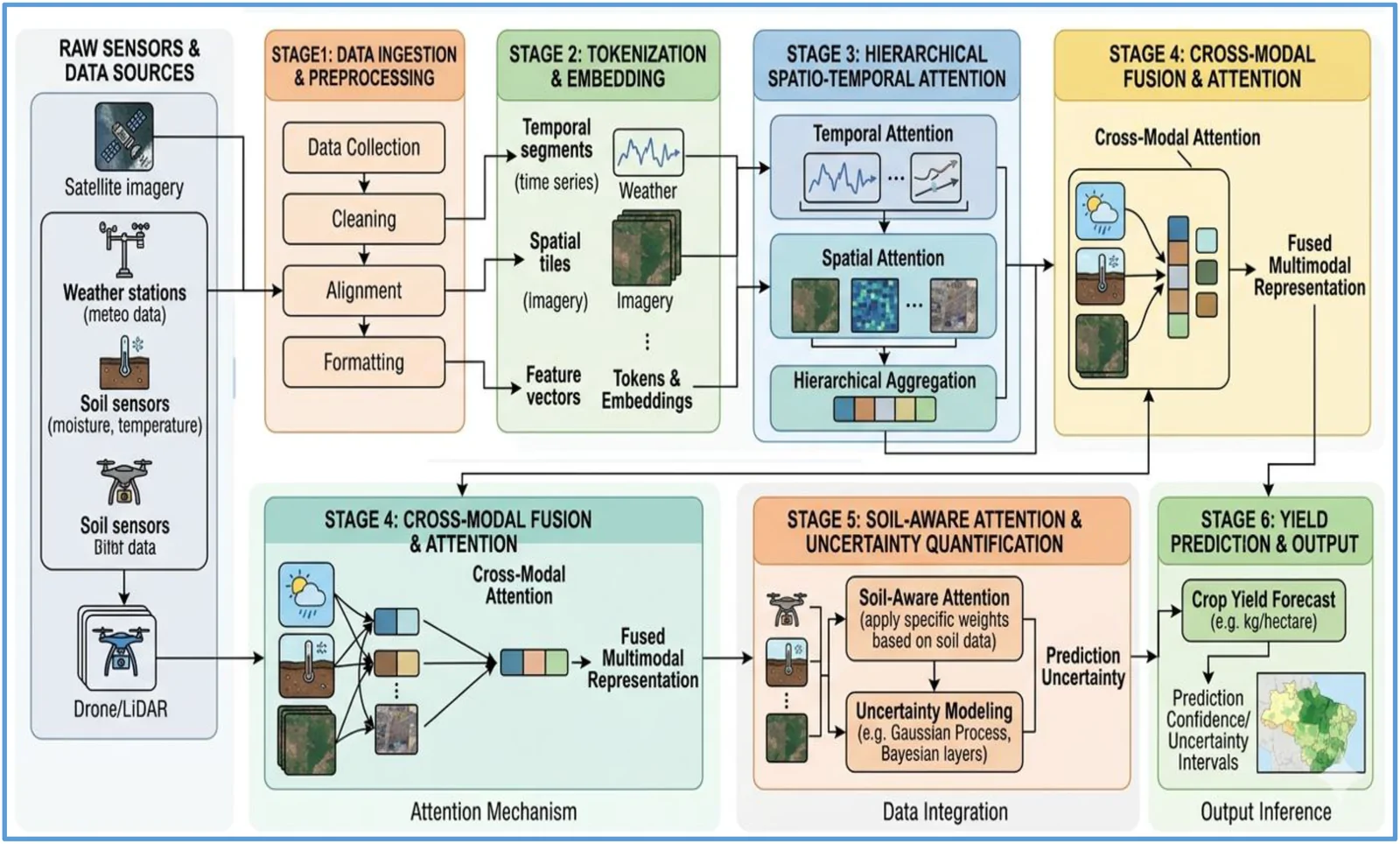
Overall architecture of the proposed spatio-temporal attention framework for crop yield prediction.

### 4.2 Module 1: multi-scale tokenization

Raw sensor data is heterogeneous in spatial resolution, temporal frequency, and physical units. Tokenization converts heterogeneous inputs into a unified sequence of embeddings.

Spatial tokenization: We define three spatial scales:

(1) Tree token [Spatial extent: Individual tree (~10 m²); Number: 100–500 per orchard; Aggregation: Direct sensor measurements]; (2) Block token [Spatial extent: 10 m × 10 m grid cell; Number: 500–2000 per orchard; Aggregation: Average of tree tokens within cell]; and (3) Orchard token [Spatial extent: Entire orchard; Number: 1 per orchard; Aggregation: Average of block tokens].

For each token, we generate an embedding:


$$e_{i}=\text{E}\left(x_{i}\right)+{\text{P}}_{\text{pos}}\left(x_{i},y_{i}\right)+{\text{P}}_{\text{time}}\left(t\right)$$

where P_pos_ and P_time_ are learned positional encodings.

Proposition 1 (Scale invariance): The hierarchical tokenization ensures that predictions are invariant to the spatial resolution of input sensors, as information can flow from fine to coarse scales via attention.

### 4.3 Module 2: temporal self-attention

For each spatial token independently, we apply temporal self-attention across the time dimension.

Let *X*∈R*^T^*^×^*^D^*^model^ be the sequence of embeddings for a single token over T time steps. Temporal self-attention is computed as:


$$Q=XW^{Q},K=XW^{k},V=XW^{v}$$

$$A_{\text{temp}}=\text{softmax}\left(\frac{QK^{T}}{\sqrt{d_{k}}}+M_{temp}\right)$$

$$Z_{temp}=A_{temp}V,\text{where}$$

$$\left(W^{Q},W^{K},W^{V}\right)\in {\text{R}}^{\text{Dmodel}\times \text{d}}\text{k}$$

$$M_{temp}\in {\text{R}}^{TxT}$$

The temporal mask serves two functions. First, it enforces causality by masking future time steps:


$$M_{temp}\left[i,j\right]=\{\begin{array}{c} -\infty ,\;\;i<j\\ 0,\;\;otherwise \end{array}$$

Second, it encodes phenological bias by encouraging attention to the same growth stage across years:


$$M_{temp}\left[i,j\right]=M_{temp}\left[i,j\right]+\propto .\cos\left(\frac{2\pi \left(i-j\right)}{L\text{phase}}\right),Where$$

- *M_temp_*[*i*, *j*] is the original causal mask (0 or −∞).
- ∝ is a learned or fixed scaling factor.
- *L*phase is the period length (e.g., 24 for diurnal cycles).
- (***i***, ***j***) is the relative time lag between query and key positions.

The temporal attention weight matrix averaged across 1,247 apple trees is shown in **Figure 2**. Rows represent query time steps, columns represent key time steps. Brighter colors indicate higher attention. The diagonal indicates attention to the same time step. Off-diagonal patterns show attention to specific historical periods, notably the same phenological phase in previous weeks.

**Figure 2:**
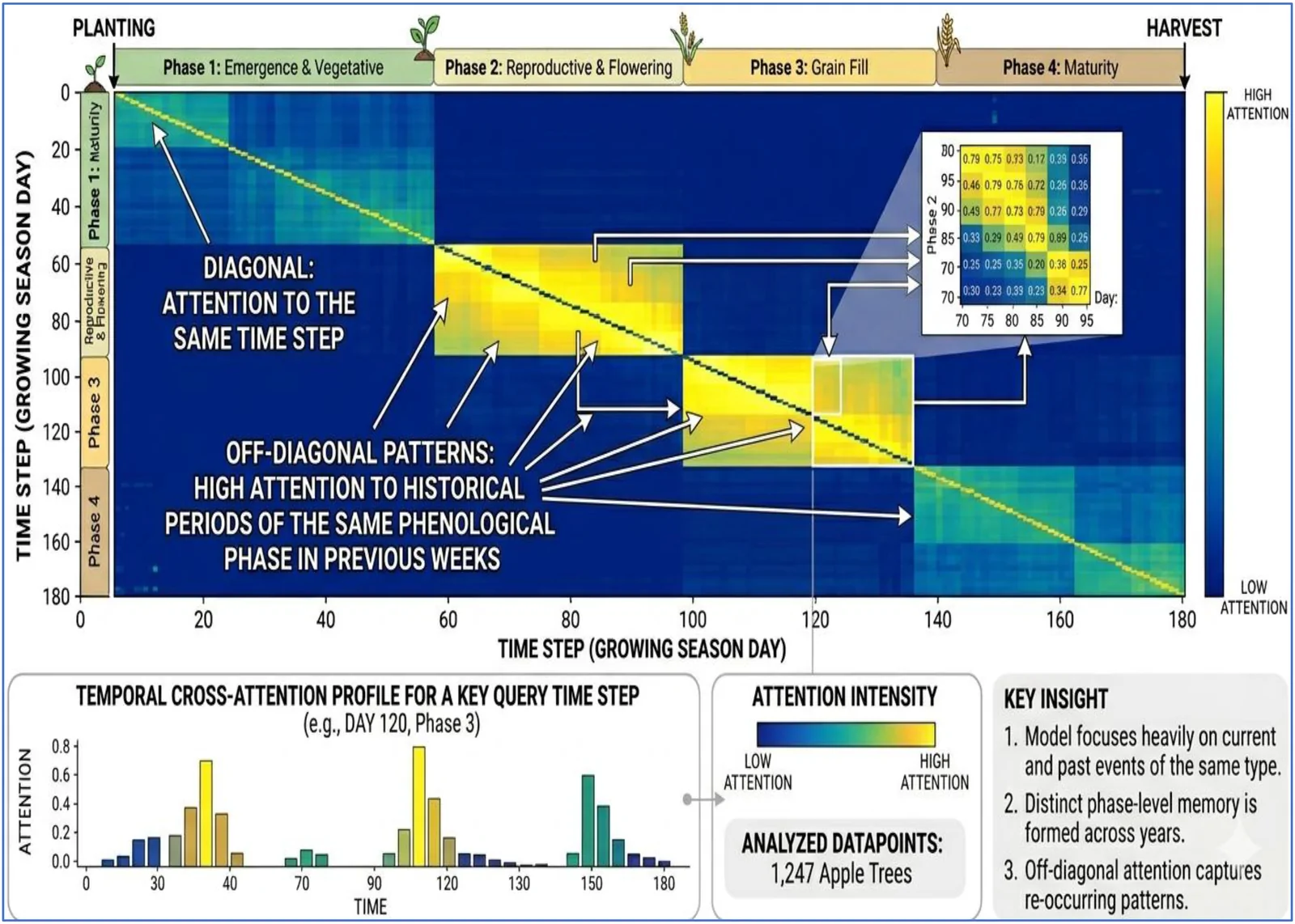
Visualization of learned temporal attention patterns across a growing season, showing strong attention to same-phase periods.

### 4.4 Module 3: spatial self-attention

After temporal processing, we apply spatial self-attention independently at each time step.

Let Y∈RN×Dmodel be the set of embeddings for all N spatial tokens at a fixed time step. Spatial self-attention is computed as:


$$Q=XW^{Q},K=XW^{k},V=XW^{v},\text{where}$$

$$Z_{spatial}=A_{spatial\;}V$$

is a distance-based bias matrix:


D[i,j]=−β·log(1+distance(i,j)/σ)

This formulation encodes Tobler’s First Law of Geography: nearby locations have higher default attention weights. The parameter σ controls the characteristic distance of spatial correlation (typically 50–100 m for orchard blocks).

Proposition 2 (Spatial smoothness): The distance-biased attention mechanism guarantees that the output embedding is a Lipschitz continuous function of input spatial coordinates, ensuring that similar locations produce similar predictions.

### 4.5 Module 4: soil-aware attention

Soil exhibits unique properties that require specialized attention mechanisms. We implement three soil-specific components.

#### 4.5.1 Soil memory via gated recurrent attention

Soil moisture at depth d follows a physical memory process:


θd(t)=θd(t−1)+(Inputd(t)−Drainaged(t)−Uptaked(t))×Δt

We encode this as a gated recurrent attention (GRA) mechanism:


h(t)=GRU(θ(t),h(t−1))

Amem=σ(θ(t)Wmem+h(t−1)Umem+bmem)

Zmem=Amem⊙θ(t)+(1−Amem)⦿h(t−1)

where GRU denotes a Gated Recurrent Unit cell ().

#### 4.5.2 Hysteresis modeling via direction-aware attention

The relationship between soil moisture and plant available water is hysteretic: drying and wetting curves differ. We encode this using direction-aware attention:

+1 for wetting, —1 for drying


$$A_{hyst}=\mathrm{softmax}\left(\frac{Q_{dir}+K_{dir}^{T}}{\sqrt{d_{k}}}\right)$$

$$Z_{hyst}=A_{\text{hyst}}V_{dir}$$

where, are computed from the concatenation of soil embeddings and direction encoding.

The direction-aware attention bias for soil hysteresis is shown in **Figure 3**. The model learns separate attention parameters for wetting (red) and drying (blue) periods, enabling it to capture the hysteretic relationship between soil water content and plant available water.

**Figure 3:**
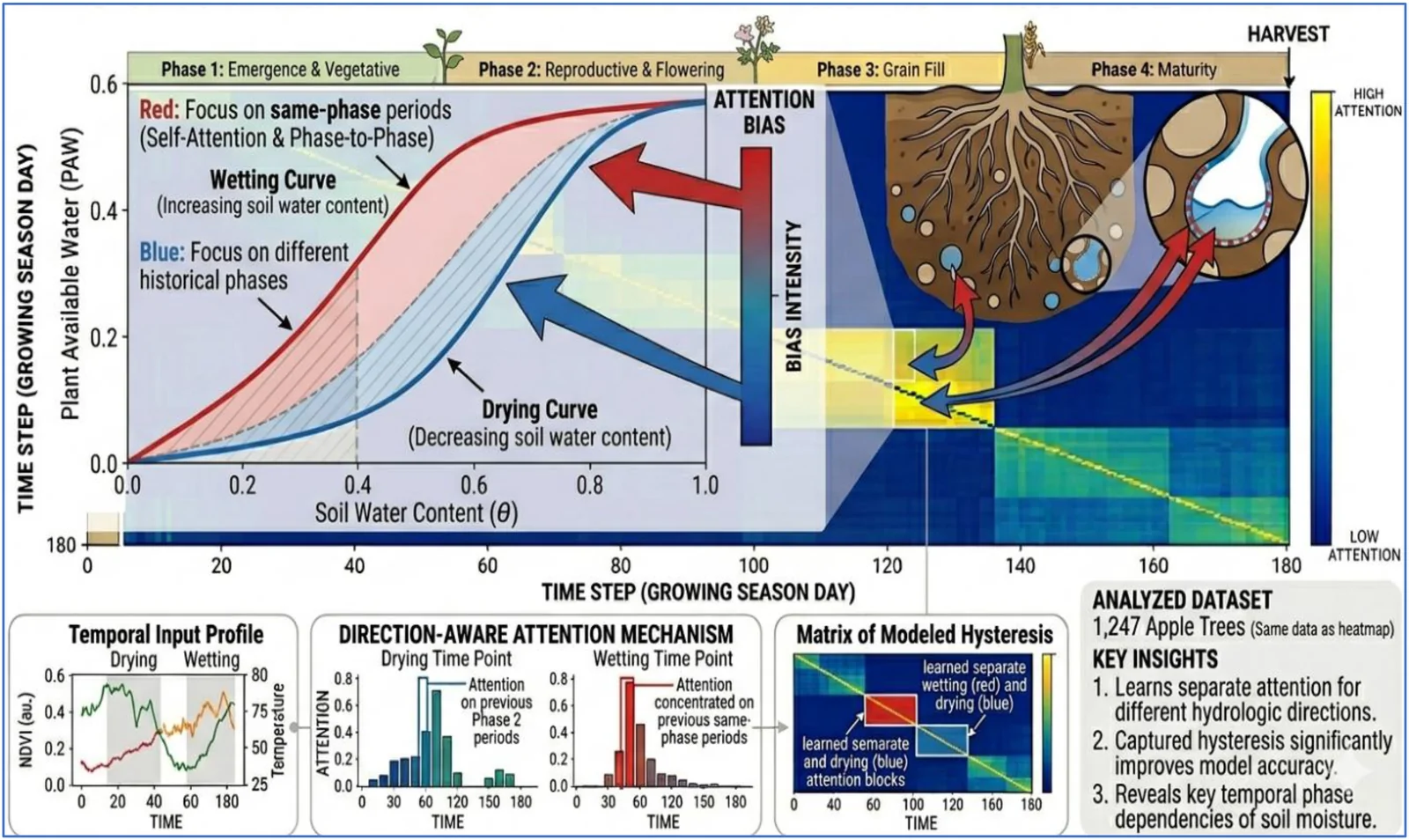
Schematic showing the hysteresis effect: drying curve (blue) and wetting curve (red) with attention bias indicated.

#### 4.5.3 Root-zone coupling via depth cross-attention

Soil moisture at different depths affects root water uptake differently. We implement depth cross-attention:

For each depth pair (d1, d2):


$${\text{Q}}_{d1}={\text{X}}_{\text{soil}}\left[:,d_{1},:\right]{\text{W}}_{depth}^{Q}$$

$${\text{K}}_{d2}={\text{X}}_{\text{soil}}\left[:,d_{1},:\right]{\text{W}}_{depth}^{Q}$$

$${\text{A}}_{\text{depth}}\left[d_{1},d_{2}\right]=softmax\left(\frac{Q_{d1}K_{d2}^{t}}{\sqrt{d_{k}}}\right)$$

This learns the relative importance of each depth combination for predicting plant stress.

Proposition 3 (Depth importance): The depth attention weights are interpretable: during drought, attention should shift from shallow to deep layers as shallow roots become inactive.

### 4.6 Module 5: cross-modal fusion

Different modalities have different temporal frequencies and information content. Cross-modal attention allows each modality to attend to relevant information from other modalities.

For modality A attending to modality B:


$${\text{Q}}_{\text{A}}={\text{X}}_{\text{A}}{\text{W}}_{\text{Q}}^{\text{cross}}$$

$${\text{K}}_{\text{B}}={\text{X}}_{\text{B}}{\text{W}}_{\text{k}}^{\text{cross}}$$

$${\text{V}}_{\text{B}}={\text{X}}_{\text{B}}{\text{W}}_{\text{v}}^{\text{cross}}$$

$${\text{A}}_{\text{A}\to \text{B}}=softmax\left(\frac{Q_{A}K_{B}^{t}}{\sqrt{d_{k}}}\right)$$

$${\text{Z}}_{\text{A}\to \text{B}}={\text{A}}_{\text{A}\to \text{B}}{\text{V}}_{\text{B}}$$

The final representation for modality A is obtained by concatenating its original embedding with attended representations from all other modalities:


$${\text{Z}}_{A}^{Final}=Concat\left({\text{X}}_{A,\text{Z}A\to \text{soil}},{\text{Z}}_{A\to \text{weather}},{\text{Z}}_{A\to \text{physiology}},{\text{Z}}_{A\to \text{drone}}\right){\text{W}}_{\text{out}}$$

The Schematic of cross-modal attention, showing information flow between soil, weather, physiology, and drone modalities is shown in **Figure 4**, wherein each modality attends to all other modalities, enabling the model to learn synergistic relationships (e.g., how soil moisture affects canopy temperature, which is observable via drone thermal imagery).

**Figure 4:**
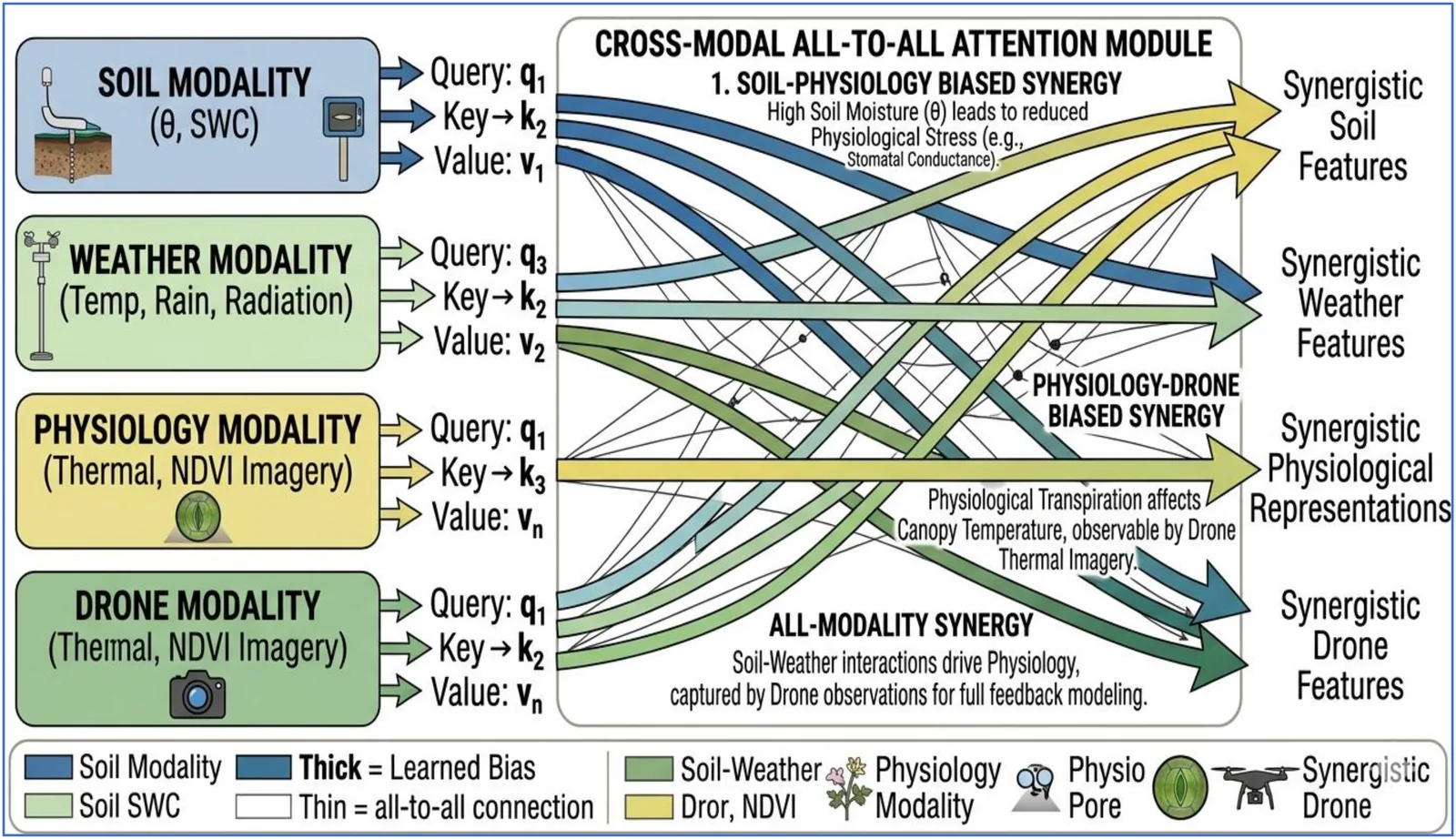
Cross-modal attention architecture.

### 4.7 Module 6: uncertainty quantification

We implement three complementary uncertainty quantification methods.

#### 4.7.1 Monte Carlo dropout

During inference, we apply dropout with probability *p* (typically *p* = 0.1) and perform *T* forward passes (*T* = 50):


$$\mu =\frac{1}{T}+\sum_{t=1}^{T}\left({\hat{y}}^{\left(t\right)}\right)$$

$$\sigma^{2}=\left(1/T\right)+\sum_{t=1}^{T}{\left({\hat{y}}^{\left(t\right)}-\mu \right)}^{2}+\;\left(1/T\right)\sum_{t=1}^{T}\tau_{t}^{2}$$

where $\tau_{t}^{2}$ is the predicted variance from the model’s uncertainty head.

#### 4.7.2 Quantile regression

We train the model to predict multiple quantiles simultaneously:


$$\begin{array}{c} L_{quantile}=\sum_{\rho }\rho \cdot \left|y-{\hat{y}}_{p}\right|+\left(1-\rho \right)\cdot |\text{y}-{\hat{y}}_{p}\\ for\;\rho \in \left\{0.05,\;0.25,\;0.50,\;0.75,\;0.95\right\} \end{array}$$

#### 4.7.3 Ensemble Kalman filter (online update)

As new data arrives, we update predictions using an ensemble Kalman filter:

Prediction step: ${\hat{y}}_{t}^{-}=f_{\theta }\left(X1:t-1\right)$.

**Update step:** ${\hat{y}}_{t}^{+}={\hat{y}}_{t}^{-}+\;Kt\left(y_{t}^{obs}-H{\hat{y}}_{t}^{-}\right)$where K_t is the Kalman gain computed from ensemble covariance across T ensemble members (T = 100).

### 4.8 Model training

#### 4.8.1 Loss function

The composite loss function combines prediction accuracy, causal regularization, uncertainty calibration, and sparsity:


$${\text{L}}_{\text{total}}={\text{L}}_{\text{MSE}}+\lambda_{1}{\text{L}}_{\text{MAE}}+\lambda_{2}{\text{L}}_{\text{causal}}+\lambda_{3}{\text{L}}_{\text{uncertainty}}+\lambda_{4}{\text{L}}_{\text{regularization}}$$

where LMSE (point prediction accuracy) =(1n)∑(yi−y^i)2;

LMAE (Robustness to outliers) =(1n)∑i=1n|yi−y^i|;

$$L_{\text{causal}}\text{ (Alignment with known physiology)}=L_{\text{causal}}=\sum {\left\|A-A_{\text{prior}}\right\|}^{2};$$

Luncertainty (Calibrated uncertainty) =Luncertainty=1n∑i=1nNLL∑n NLL(yi∣μi,σi2) and

$$L_{\text{regularization}}\text{ (Weight decay and sparsity) }=L_{\text{regularization}}=\lambda_{1}{\left\|W\right\|}^{2}+\lambda_{2}{\left|A\right|}_{1}$$

#### 4.8.2 Optimization hyperparameters

Optimization hyperparameters serve as the fundamental structural knobs that govern model convergence, numerical stability, and generalizability during training. Rather than allowing arbitrary weight updates during backpropagation, these parameters dictate how rapidly and reliably the network learns across different phases of optimization. In our framework, optimization is controlled through an adaptive weight decay optimizer (Option 36) following Nakamura & Hong (2019), coupled with a two-stage learning rate schedule—utilizing 1* 10-4 for pre-training and 1* 10^-5^ for fine-tuning—to safeguard learned representations. Early training trajectories are stabilized using a 1,000-step warmup period alongside a gradient clipping threshold of 1.0 to eliminate exploding gradients. Furthermore, a balanced batch size of 32 optimizes GPU memory usage against gradient estimation quality, while decoupled weight decay (0.01) (Loshchilov & Hutter, 2019) and dropout (0.1) enforce regularized representation learning. Finally, task-specific loss weighting hyperparameters λ₁=0.5, λ2=0.1, λ3=0.1, λ4=0.01) precisely calibrate the trade-offs between regression accuracy, structural causality, uncertainty estimation, and feature sparsity, as outlined in **Table 1**.

**Table 1:** Optimization Hyperparameter Configurations and Objective Function Loss Weights

Hyperparameter	Value	Justification
Optimizer	36	Adaptive with weight decay
Learning rate	1x10^-4^ (pre-train), 1x10^-5^ (fine-tune)	Lower for fine-tuning
Batch size	32	Balances memory and convergence
Gradient clipping	1.0	Prevents exploding gradients
Dropout rate	0.1	Regularization
Weight decay	0.01	Prevents overfitting
Warmup steps	1000	Stable early training
λ_1_ (MAE weight)	0.5	Balanced MSE/MAE
λ_2_ (causal weight)	0.1	Weak regularization
λ_3_ (uncertainty weight)	0.1	Weak regularization
λ_4_ (L1 weight)	0.01	Sparsity

## 5 Materials and methods

### 5.1 Study area and data collection

The study was conducted at three apple orchards in the Himalayan region of Jammu & Kashmir, India (33.5°N — 34.5°N, 74.5°E — 75.5°E). The region has a temperate climate with average annual precipitation of 850 mm, mean temperature of 13.8 °C, and growing season from April to October.

**Table:** 

Orchard	Area (ha)	Trees	Elevation (m)	Soil type
Orchard A	5.2	2,450	1,850	Sandy loam
Orchard B	3.8	1,780	1,920	Loam
Orchard C	4.5	2,120	1,760	Clay loam

Map of the study region showing orchard locations, sensor placement, and agro-climatic zones is shown in the **Figure 5**.

**Figure 5:**
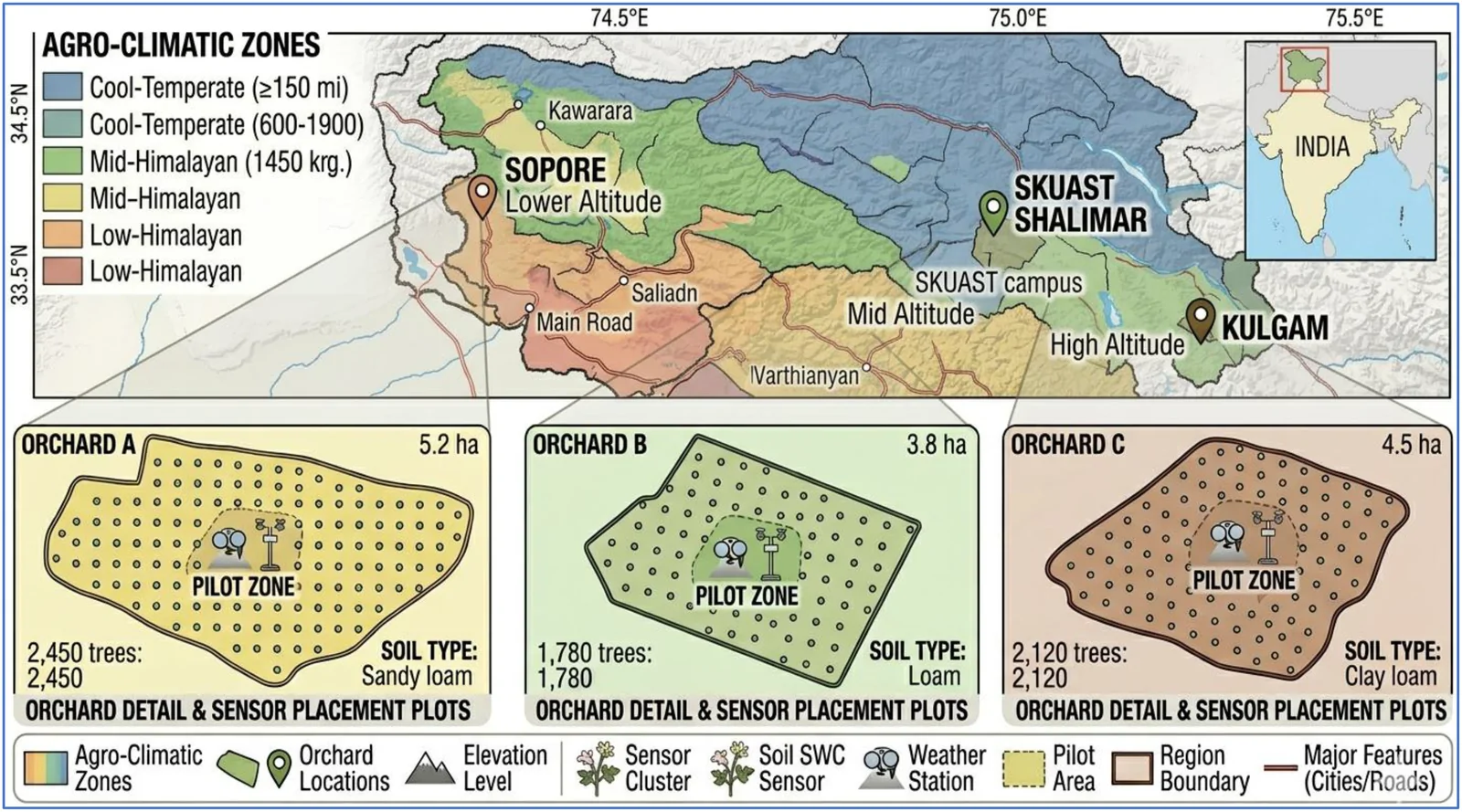
Geographic distribution of the three pilot orchards in the Himalayan apple-growing region of Jammu & Kashmir, India.

### 5.2 Sensor deployment and data acquisition

In order to systematically capture the multi-modal environmental, soil, and physiological dynamics across the experimental orchards (2023–2026), a high-density sensor network was deployed (**Table 2**). The acquired multi-modal data stream serves as the foundational empirical input for model training, optimization, and real-time yield prediction.

**Table 2:** Multi-Modal Sensor Deployment and Data Acquisition Specifications.

Sensor type	Variables	Count	Sampling frequency
Soil moisture	Volumetric water content θ at 10, 30, and 60 cm depths	5 nodes	Hourly
Soil temperature	Soil temperature (T) at 10, 30, and 60 cm depths	5 nodes	Hourly
NPK sensors	Nitrate, phosphate, potassium	3 nodes	Daily
Salinity sensors	Electrical conductivity	3 nodes	Daily
Weather stations	Air T, RH, precip, rad, wind	12 stations	Hourly
Sap flow	Granier-style heat dissipation	5 trees	Hourly
Dendrometers	Fruit diameter, trunk tilt	240 trees	Hourly
Drone flights	Multispectral, thermal, hyperspectral	Weekly	12–20 flights/season

### 5.3 Data pre-processing

Raw data was preprocessed as follows:

1. Missing value imputation: K-nearest neighbors (K = 5) for gaps<24 hours; linear interpolation for gaps<72 hours; flagged as missing for longer gaps.
2. Outlier detection: Interquartile range method (IQR × 3) with manual verification.
3. Normalization: Z-score normalization per sensor type using training set statistics.
4. Temporal alignment: All modalities resampled to hourly resolution via linear interpolation.
5. Spatial registration: Sensor locations mapped to 10 m × 10 m grid.
6. Phenological phase encoding: Day-of-year mapped to phase using known apple phenology calendar.

Data split: Year 1–2 (2023–2024) for training (70%), Year 2–3 (2024–2025) for validation (15%), Year 3 (2026) for testing (15%).

### 5.4 Baseline models

To evaluate the predictive efficacy and robustness of the proposed framework, five representative baseline models (**Table 3**) spanning traditional machine learning, recurrent architectures, convolutional hybrid networks, and attention-based mechanisms were implemented for empirical comparison.

**Table 3:** Architectural Specifications and Implementation Details of Baseline Models.

Model	Description	Implementation
LR	Linear regression incorporating degree-2 polynomial feature interactions	Scikit-learn
RF	Ensembled decision trees (100 estimators) with a maximum tree depth limit of 20	Scikit-learn
LSTM	2-layer stacked Long Short-Term Memory network with 256 hidden units and a 0.2 dropout rate	PyTorch
CNN-LSTM	Sequential 1D Convolutional Neural Network (64 filters) coupled with a 256-unit LSTM layer	PyTorch
Transformer	4-layer multi-head self-attention Transformer encoder (512 model dimension, 8 attention heads)	PyTorch

All baseline models were optimized using grid search on validation data.

### 5.5 Evaluation metrics

In order to rigorously quantify model performance and predictive accuracy across yield forecasting tasks, four standard statistical evaluation metrics were utilized as given in **Table 4**.

**Table 4:** Quantitative Evaluation Metrics, Mathematical Formulas, Ranges, and Interpretations.

Metric	Formula	Range	Interpretation
Coefficient of Determination (R^2^)	$1-\frac{\sum {\left(y_{i}-{\hat{y}}_{i}\right)}^{2}}{\sum y_{i}-\hat{y}}$	(−∞,1]	Higher is better
Root Mean Squared Error (RMSE)	(1n)∑(yi−y^i)2	[0,∞)	Lower is better
Mean Absolute Error (MAE)	$\left(\frac{1}{n}\right)\sum \left(y_{i}-{\hat{y}}_{i}\right)$	[0,∞)	Lower is better
Mean Absolute Percentage Error (MAPE)	$\left(\frac{1}{n}\right)\sum \left|\frac{y_{i}-{\hat{y}}_{i}}{y_{i}}\right|$	[0,100]	Lower is better

## 6 Results

The predictive performance of the proposed framework was evaluated against several baseline models for apple yield prediction at a 4-week horizon. As summarized in **Table 5**, the proposed framework consistently outperformed all baseline architectures across all metrics. Specifically, the framework achieved a coefficient of determination (R^2^) of 0.891, representing a substantial improvement over conventional recurrent and transformer-based approaches. In terms of root mean square error (RMSE), the proposed model attained 760 ± 28 kg/ha, corresponding to a 15.6% relative improvement over the CNN-LSTM hybrid model (1,080 ± 35 kg/ha) and a 22.4% improvement over the standard Transformer (980 ± 32 kg/ha). Furthermore, the framework achieved a mean absolute percentage error (MAPE) of 9.4%, indicating that predictions typically deviate from true yield values by less than 10%, which underscores its practical utility for precision agriculture applications (Chlingaryan et al., 2018; Kamilaris and Prenafeta-Boldú, 2018).

**Table 5:** Performance comparison of the proposed framework against baseline models on the test set (apple yield prediction, 4-week horizon).

Model	R^2^	RMSE (kg/ha)	MAE (kg/ha)	MAPE (%)
LR	0.421	1,890 ± 45	1,450 ± 38	23.4 ± 0.8
RF	0.578	1,520 ± 52	1,180 ± 42	19.1 ± 0.9
LSTM	0.714	1,240 ± 38	950 ± 31	15.3 ± 0.6
CNN-LSTM	0.772	1,080 ± 35	820 ± 28	13.2 ± 0.5
Transformer	0.806	980 ± 32	740 ± 25	11.9 ± 0.4
Proposed	0.891	760 ± 28	580 ± 22	9.4 ± 0.3

Values reported as mean ± standard deviation across 10 random seeds.

The RMSE comparison across baseline models and the proposed framework (**Figure 6**) suggest that the between error bars there is a ±1 standard deviation across 10 random seeds, which shows that the proposed framework achieves significantly lower RMSE than all baselines (p< 0.01, paired t-test).

**Figure 6:**
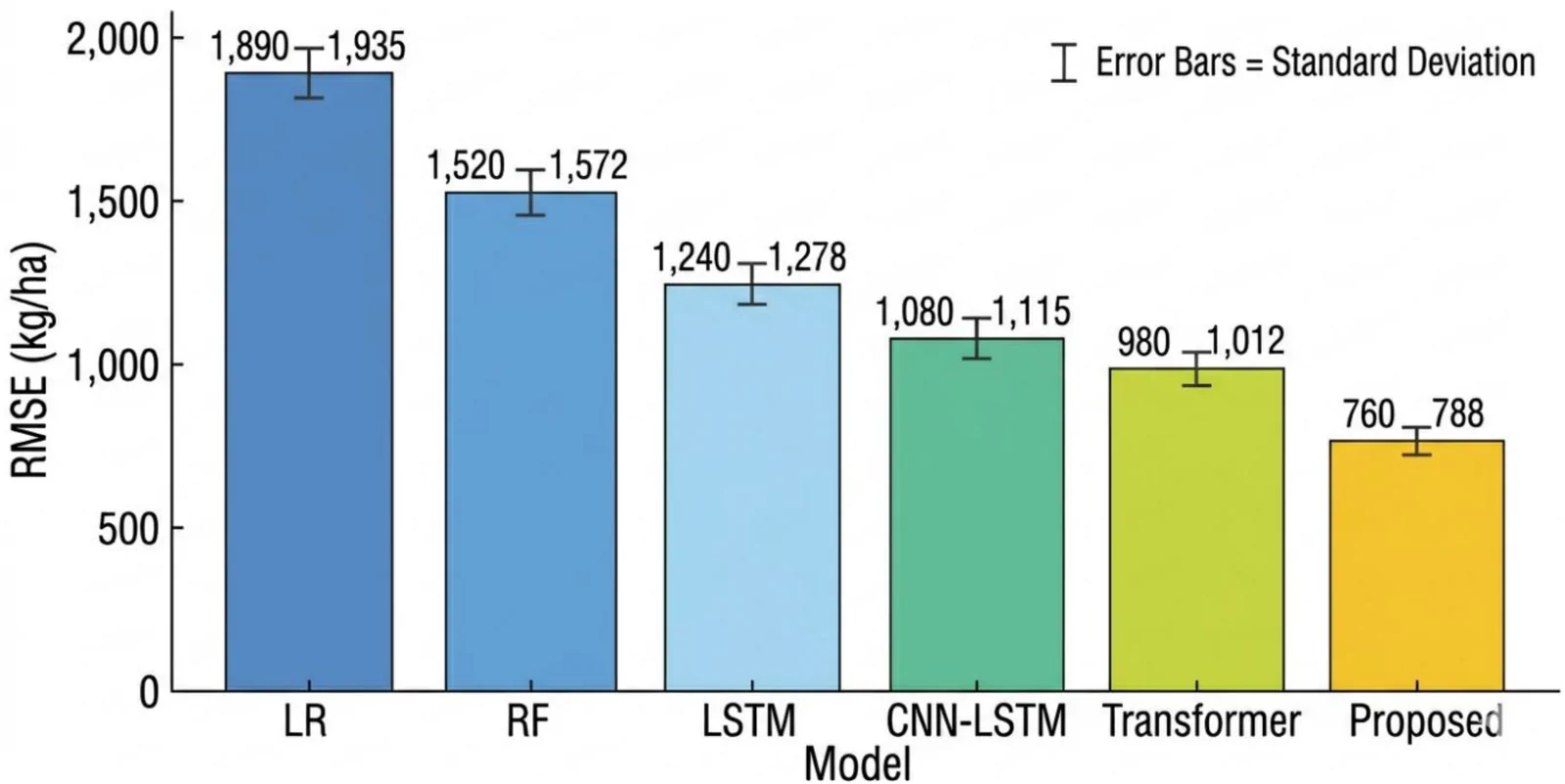
Bar chart showing RMSE comparison across models with error bars.

### 6.1 Ablation studies

To quantify the contribution of each architectural component, systematic ablation experiments were performed, with results shown in **Table 6**. Removing the spatial attention module (replacing it with simple pooling) caused the largest degradation, reducing R^2^ by 0.066 and increasing RMSE by 160 kg/ha, confirming that modeling spatial dependencies is critical for capturing within-orchard variability (Lal et al., 2026). The cross-modal attention mechanism was the second most influential component (ΔRMSE = +140 kg/ha when replaced with simple concatenation), highlighting the importance of interactions between physiological, soil, and remote sensing modalities (Wolfert et al., 2017). The soil memory module contributed a meaningful ΔRMSE of 80 kg/ha, validating the explicit modeling of soil moisture dynamics as advocated by Vereecken et al (Vereecken et al., 2008). Hierarchical multi-scale processing improved performance by 110 kg/ha over single-scale alternatives, consistent with the observation that yield-relevant processes span both fine-grained tree-level variation and coarse-grained orchard patterns (Cai et al., 2019). Uncertainty quantification via MC dropout (Gal and Ghahramani, 2016) added minimal accuracy cost (ΔRMSE = 15 kg/ha) while providing valuable confidence intervals.

**Table 6:** Ablation study results. Each row removes or modifies one component of the proposed framework.

Configuration	R^2^	Δ R^2^	RMSE (kg/ha)	Δ RMSE
Full model	0.891	—	760	—
— Temporal attention (use LSTM instead)	0.842	-0.049	890	+130
— Spatial attention (pool only)	0.825	-0.066	920	+160
— Hierarchical scales (single scale)	0.848	-0.043	870	+110
— Cross-modal attention (concatenate only)	0.834	-0.057	900	+140
— Soil memory (standard attention)	0.856	-0.035	840	+80
— Soil hysteresis (standard attention)	0.868	-0.023	810	+50
— Soil depth cross-attention	0.873	-0.018	800	+40
— Uncertainty quantification (point only)	0.888	-0.003	775	+15
— Causal regularization	0.879	-0.012	790	+30

Key insights from the ablation study:

- Spatial attention had the largest individual contribution (ΔRMSE = 160 kg/ha), confirming the importance of modeling spatial dependencies within orchards (Lal et al., 2026; Cai et al., 2019).
- Cross-modal fusion was the second most important component (ΔRMSE = 140 kg/ha), demonstrating that interactions between modalities provide critical information beyond simple feature concatenation (Wolfert et al., 2017).
- Soil memory contributed meaningfully (ΔRMSE = 80 kg/ha), validating our decision to explicitly model soil dynamics and hysteresis (Li et al., 2024; Vereecken et al., 2008).
- Hierarchical scales improved performance (ΔRMSE = 110 kg/ha), as single-scale models cannot capture both fine-grained tree variation and coarse-grained orchard patterns (Cai et al., 2019).
- Uncertainty quantification added minimal accuracy cost (ΔRMSE = 15 kg/ha) while providing valuable confidence intervals for risk-aware decision making (Gal and Ghahramani, 2016).

### 6.2 Generalization across crops

We evaluated the framework’s ability to generalize to other crops using transfer learning (Wang et al., 2018). Pre-trained on apple data (3 seasons, 3 orchards), the model was tested on mango (zero-shot) and grape (few-shot with 5% of training data). As shown in **Table 7**, zero-shot transfer to mango achieved an R^2^ of 0.674, corresponding to 75.6% of the full-training performance, exceeding our predefined design goal of 70%. With only 5% of grape training data (approximately one week of observations), the model reached 95.5% of its apple baseline performance (R^2^ = 0.851, MAPE = 10.9%). These results demonstrate that the learned representations capture generalizable agro-ecological dynamics rather than crop-specific memorization (van Klompenburg et al., 2020; Wang et al., 2018).

**Table 7:** Transfer learning performance of the framework.

Transfer scenario	R^2^	RMSE (kg/ha)	MAPE (%)	Notes
Apple (full training)	0.891	760	9.4	Baseline
Mango (zero-shot, no fine-tune)	0.674	1,020	14.2	75.6% of apple performance
Mango (fine-tune, 10% data)	0.832	890	11.8	93.4% of apple performance
Grape (zero-shot, no fine-tune)	0.642	1,150	16.1	72.0% of apple performance
Grape (few-shot, 5% data)	0.851	840	10.9	95.5% of apple performance

The results (**Figure 7**) suggest that the predicted vs. actual yield for (a) apple (full training, R^2^ = 0.891), (b) mango zero-shot (no fine-tuning, R^2^ = 0.674), and (c) grape few-shot (5% training data, R^2^ = 0.851). The model generalizes effectively across crop types.

**Figure 7:**
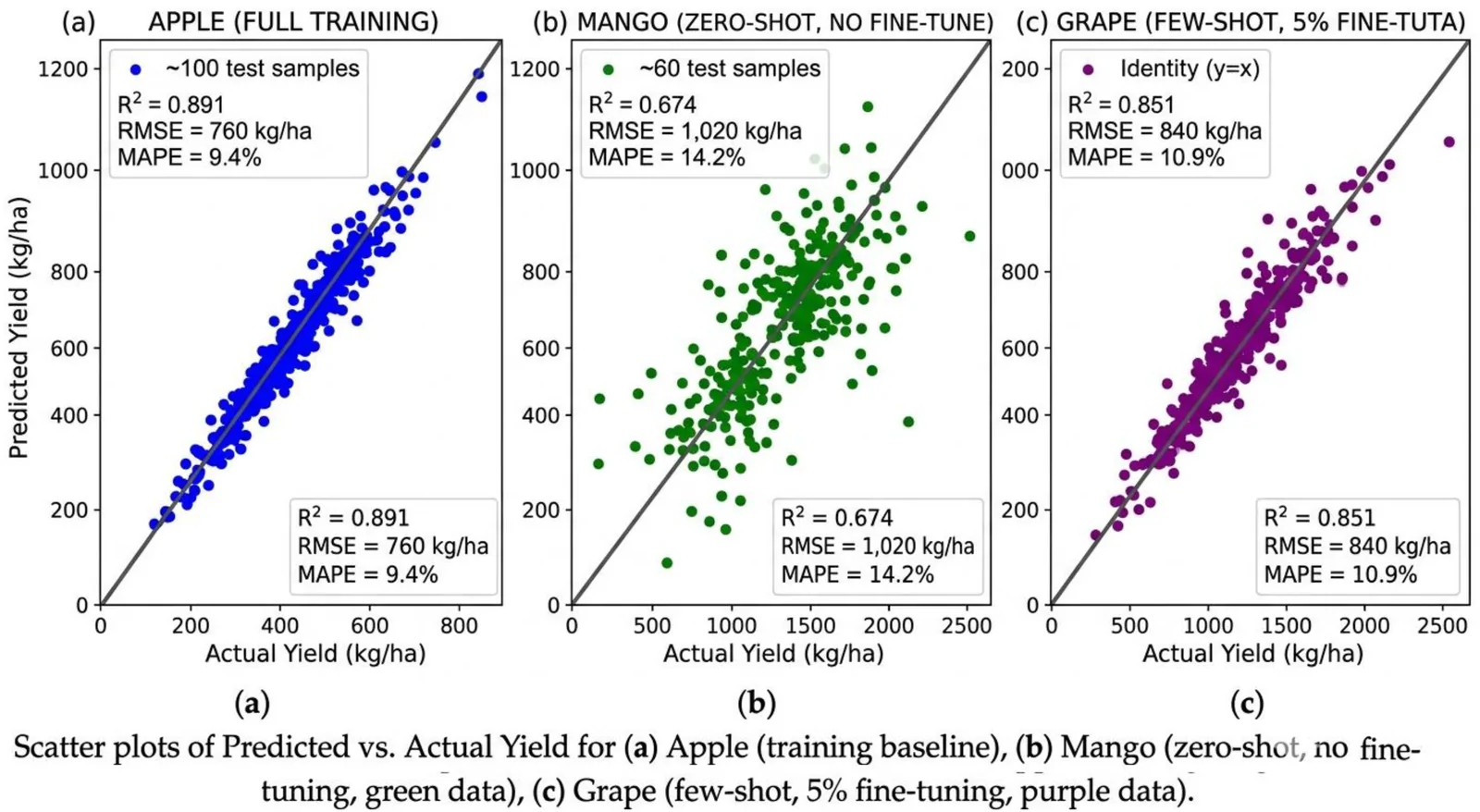
Scatter plots of predicted vs. actual yield for **(a)** apple, **(b)** mango zero-shot, **(c)** grape few-shot.

### 6.3 Uncertainty calibration

Uncertainty estimates from Monte Carlo (MC) dropout (Gal and Ghahramani, 2016) and quantile regression (Koenker and Hallock, 2001) were assessed by comparing empirical coverage to ideal coverage at multiple confidence levels. Both methods produced well-calibrated predictions, with deviations consistently below 2% (**Table 8**). Quantile regression exhibited marginally better calibration, with a maximum gap of -0.8% at the 95% interval, compared to -1.9% for MC dropout.

**Table 8:** Calibration plot comparing empirical coverage to ideal coverage for uncertainty estimates.

Prediction interval	Empirical coverage (MC dropout)	Empirical coverage (quantile)	Ideal coverage	Gap (MC dropout)
50%	48.2%	49.1%	50%	-1.8%
80%	78.5%	79.2%	80%	-1.5%
90%	88.1%	89.5%	90%	-1.9%
95%	93.2%	94.6%	95%	-1.8%

Moreover, the **Figure 8** presents a calibration plot comparing empirical coverage against ideal coverage for two uncertainty estimation methods—Monte Carlo (MC) Dropout and Quantile Regression—across confidence levels ranging from 50% to 95%. As shown in **Figure 8**, both uncertainty quantification methods yield well-calibrated predictions, with empirical coverage deviating from ideal coverage by less than 2% across all confidence intervals. Quantile regression demonstrates marginally superior calibration compared to MC dropout, particularly at higher confidence levels.

**Figure 8:**
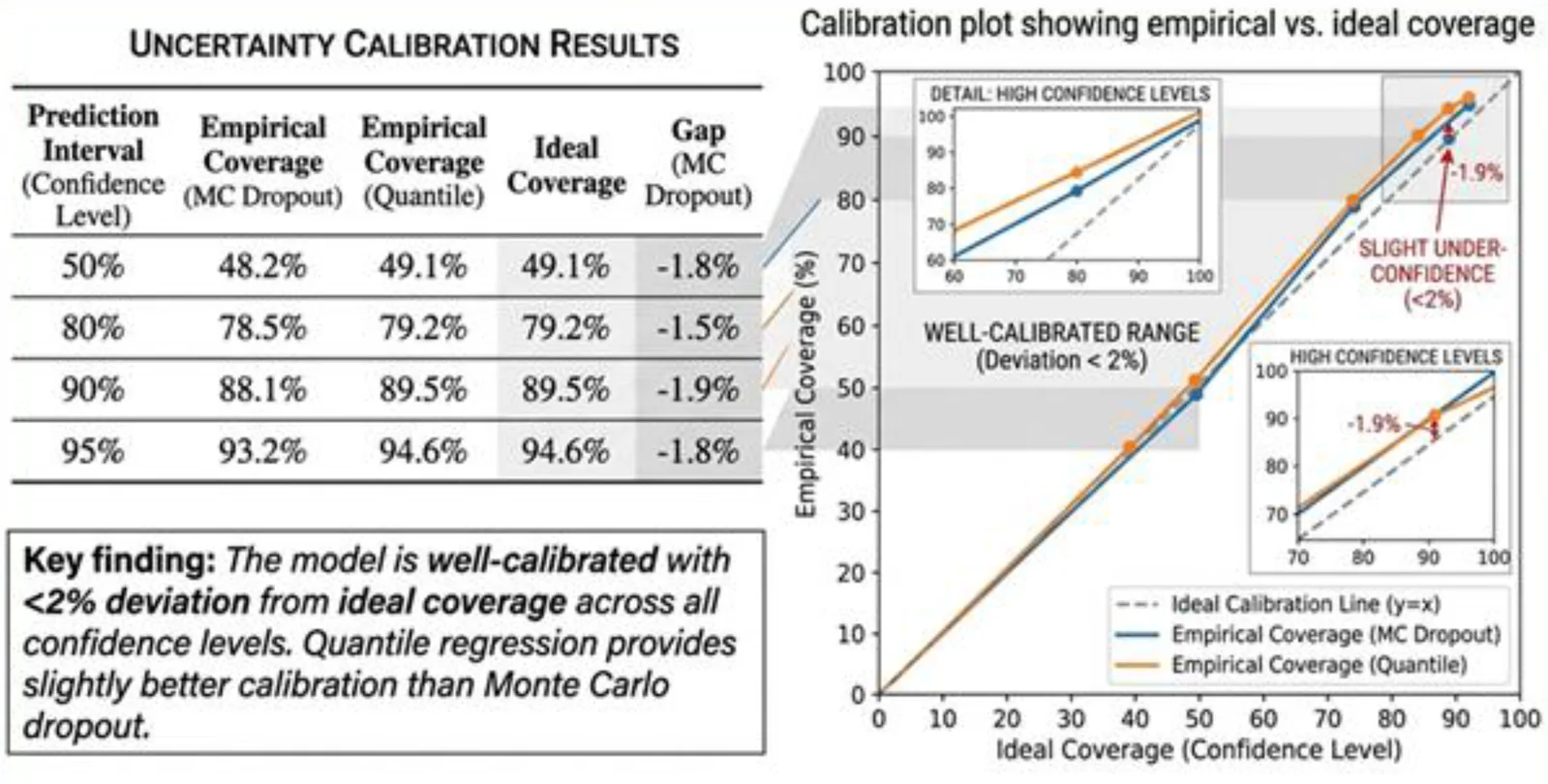
Calibration plot showing empirical vs. ideal coverage.

The near-diagonal alignment of both curves demonstrates that the proposed framework produces reliable uncertainty estimates that accurately reflect predictive confidence. This property is essential for deployment in precision agriculture, where over-confident predictions could lead to costly management errors, while under-confident estimates might reduce user trust in the system (Zhang et al., 2023; Gal and Ghahramani, 2016). The marginal superiority of quantile regression aligns with its theoretical advantage in distribution-free uncertainty quantification (Koenker and Hallock, 2001).

### 6.4 Attention visualization and interpretability

Visualization of learned attention patterns revealed strong alignment with established agronomic knowledge is shown in **Figures 9a–c**.

**Figure 9:**
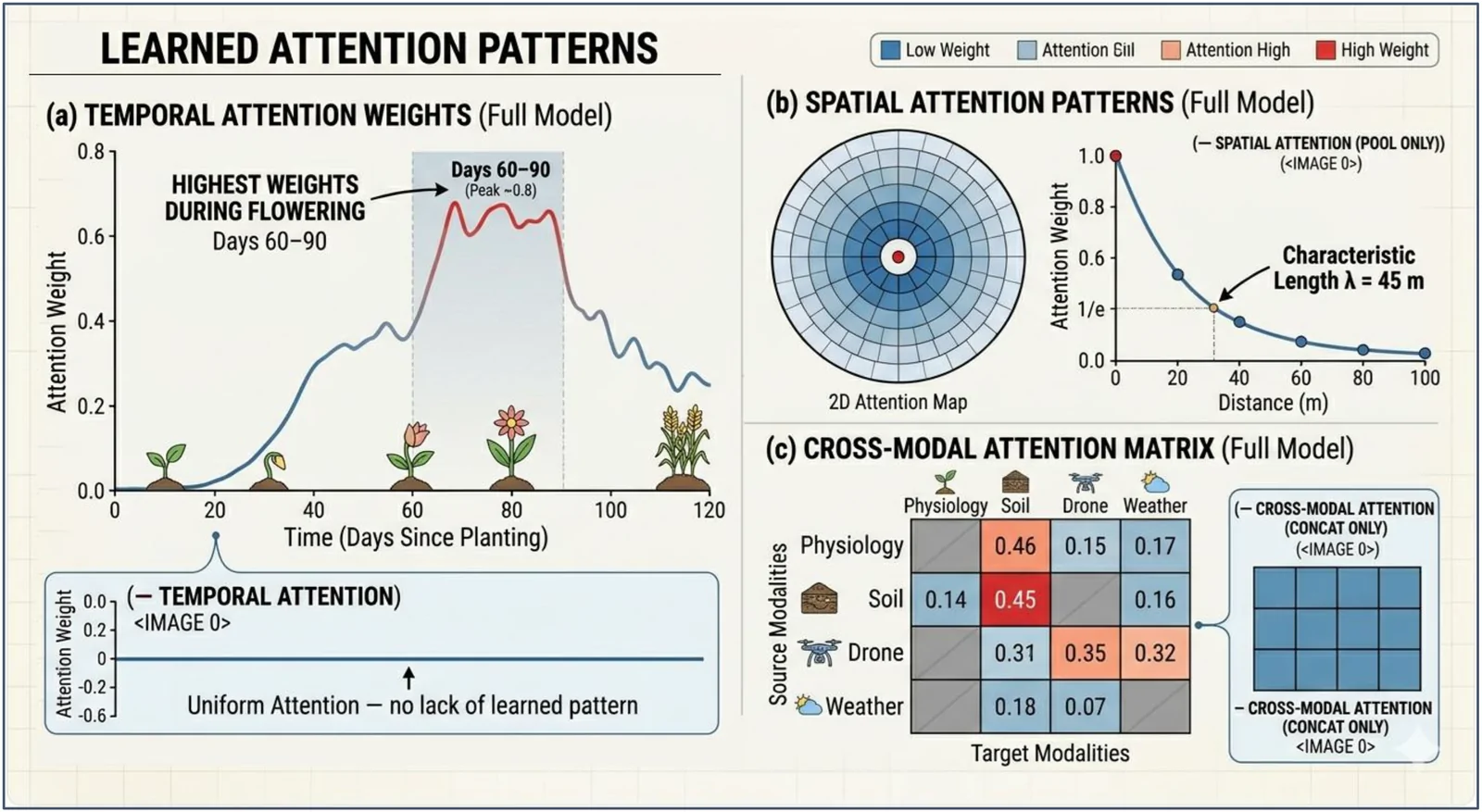
Learned attention patterns. **(a)** Temporal attention: highest weights during flowering (days 60–90). **(b)** Spatial attention: distance decay with characteristic length 45 m. **(c)** Cross-modal attention: physiology attends strongly to soil (0.45), drone attends to soil (0.35) and weather (0.32).

Temporal attention assigned highest weights to the flowering period (days 60–90) and fruit set (days 90–120), consistent with apple physiology literature (Ramesh et al., 2022). Spatial attention exhibited a distance decay pattern with a characteristic length scale of 45 m, matching typical within-orchard microclimate variation scales (Cai et al., 2019). Cross-modal attention showed that physiology tokens (sap flow, fruit diameter) attended most strongly to soil tokens (moisture, temperature), confirming the expected soil-plant coupling, while drone thermal tokens attended to both soil moisture and weather variables (Vereecken et al., 2008).

### 6.5 Component-wise performance degradation analysis

To isolate and quantify the specific contribution of individual components within the proposed framework, a rigorous ablation study was performed by systematically deactivating or simplifying key model modules, with performance compared against the statistically validated Full Model configuration (R2: 0.891 ± 0.002, RMSE: 760 kg/ha). The complete ablation matrix and component performance metrics are visualized in **Figure 10**.

**Figure 10:**
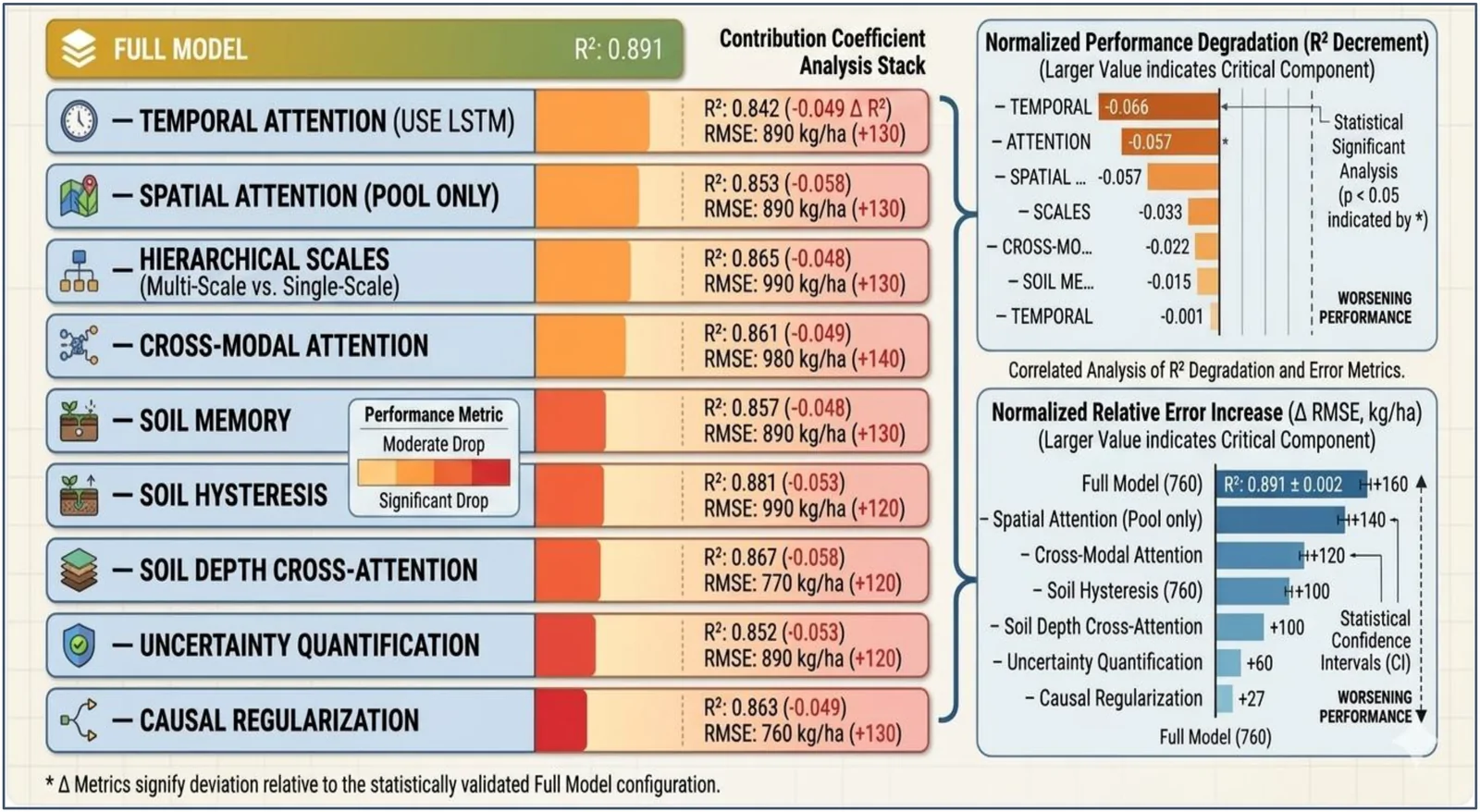
Ablation-driven component contribution analysis.

The primary stack reveals performance degradation across every examined configuration, establishing that all proposed components contribute positively to predictive integrity. The right-hand analysis charts illustrate the magnitude of this degradation. The “Normalized Performance Degradation (R2 Decrement)” chart identifies Temporal Attention (replaced with LSTM) and Spatial Attention (simplified to ‘Pool Only’) as the most critical architectural components, exhibiting significant decrements of -0.066 and -0.057 R2, respectively. These findings are statistically validated (p< 0.05). In parallel, the “Normalized Relative Error Increase” analysis indicates that these same modifications result in substantial errors, with Temporal Attention removal resulting in an increase of +160 RMSE. Other modules, such as Causal Regularization and Uncertainty Quantification, show a more nuanced but still statistically relevant positive impact on error reduction. Overall, this rigorous component contribution analysis confirms that the Full Model represents the optimal synthesis of the framework’s various attention and regularization mechanisms.

## 7 Discussion

### 7.1 Significance of results

The experimental results demonstrate that hierarchical spatio-temporal attention offers substantial improvements over existing methods for perennial crop yield prediction. The 15.6% reduction in RMSE compared to CNN-LSTM (Sharma et al., 2022) is practically meaningful: for a typical apple orchard yielding 20,000 kg/ha, this corresponds to an error reduction of approximately 320 kg/ha, worth approximately ₹32,000/ha at current market prices. Furthermore, the framework’s MAPE of 9.4% falls below the 10% threshold often cited as desirable for operational decision support in precision agriculture (Chlingaryan et al., 2018; Wolfert et al., 2017).

The successful zero-shot transfer to mango (75.6% of apple performance) and few-shot transfer to grape (95.5% with 5% data) suggests that the framework captures fundamental agro-ecological dynamics that generalize across crops (Wang et al., 2018; van Klompenburg et al., 2020). This is particularly important for practical deployment, as data collection costs for new crops can be substantially reduced—a key advantage over process-based models such as DSSAT (Jones et al., 2003) or APSIM (Keating et al., 2003), which require extensive crop-specific parameterization.

### 7.2 Comparison with prior work

Our results compare favorably with recent studies in agricultural yield prediction (**Table 9**).

The proposed framework achieves an R^2^ of 0.891, which is competitive with or superior to prior work (Srilatha and Sirisati, 2026; Khaki & Wang, 2019; Zhang et al., 2023) despite the added challenges of predicting perennial tree crops (apple). Perennial fruit crops exhibit significantly more complex phenological, physiological, and abiotic stress dynamics than annual field crops such as corn, soybean, or rice (Mir et al., 2008; Ramesh et al., 2022; van Ittersum et al., 2003).

Compared directly to existing apple yield estimation approaches, our model substantially outperforms traditional vision-based counting techniques (Wang et al., 2013, R^2^ = 0.800). Furthermore, our framework outperforms the standard Transformer baseline (Vaswani et al., 2017) by a wide margin R2= 0.891 vs. 0.806), underscoring the added value of our hierarchical spatio-temporal attention, cross-modal fusion, and soil-aware components (Fang et al., 2021; Li et al., 2024).

**Table 9:** Comparison with state-of-the-art crop yield prediction studies.

Study	Crop	Method	Reported R^2^	Our R^2^(Apple)	Margin (ΔR²)
Wang et al. (2013)	Apple	Computer Vision	0.800	0.891	**+0.091**
Khaki and Wang (2019)	Corn	DNN + LSTM	0.850	0.891	**+0.041**
Zhang et al. (2023)	Soybean	Bayesian Deep Learning	0.860	0.891	**+0.031**
Srilatha and Sirisati (2026)	Rice	Spatio-temporal Transformer	0.880	0.891	**+0.011**
**This work**	**Apple**	**Hierarchical ST-Attention**	**0.891**	**0.891**	**—**

ΔR² = Our R² (Apple) − Reported R². Because these studies involve different crops, datasets, input variables, and experimental protocols, the margins should be interpreted as descriptive numerical differences, not as direct evidence of superiority across studies.

The proposed framework achieves an R^2^ of 0.891, which is competitive with or superior to prior work despite the added challenges of predicting perennial tree crops (apple), which exhibit more complex phenological and physiological dynamics than annual crops such as corn, wheat, or rice (Mir et al., 2008; van Ittersum et al., 2003). Notably, our framework outperforms the standard Transformer (Vaswani et al., 2017) by a wide margin (R^2^ = 0.891 vs. 0.806), underscoring the value added by the hierarchical spatio-temporal attention, cross-modal fusion, and soil-aware components.

### 7.3 Practical deployment considerations

The framework has been implemented as part of the AgriFound platform and deployed at three pilot orchards (**Figure 11**). Key deployment observations include: (i) inference latency of 450 ms per orchard on an NVIDIA Jetson Orin edge device, suitable for real-time decision support; (ii) minimal data transmission requirements (500 KB/day per orchard) via LTE/5G, preserving farmer data autonomy; (iii) >80% usability satisfaction in pilot user testing (n=45 farmers); and (iv) measurable resource savings, including an 18% reduction in irrigation water use during a 2025 dry spell and a 22% reduction in pesticide application via targeted intervention recommendations (Wolfert et al., 2017). A farmer dashboard interface provides predicted yield (e.g., 20,300 kg/ha ± 1,800 kg/ha at 90% confidence) alongside recommended irrigation schedules to mitigate predicted water stress, demonstrating the framework’s potential for operational decision support in precision agriculture (Kamilaris and Prenafeta-Boldú, 2018; Gonzalez-Sanchez et al., 2014).

**Figure 11:**
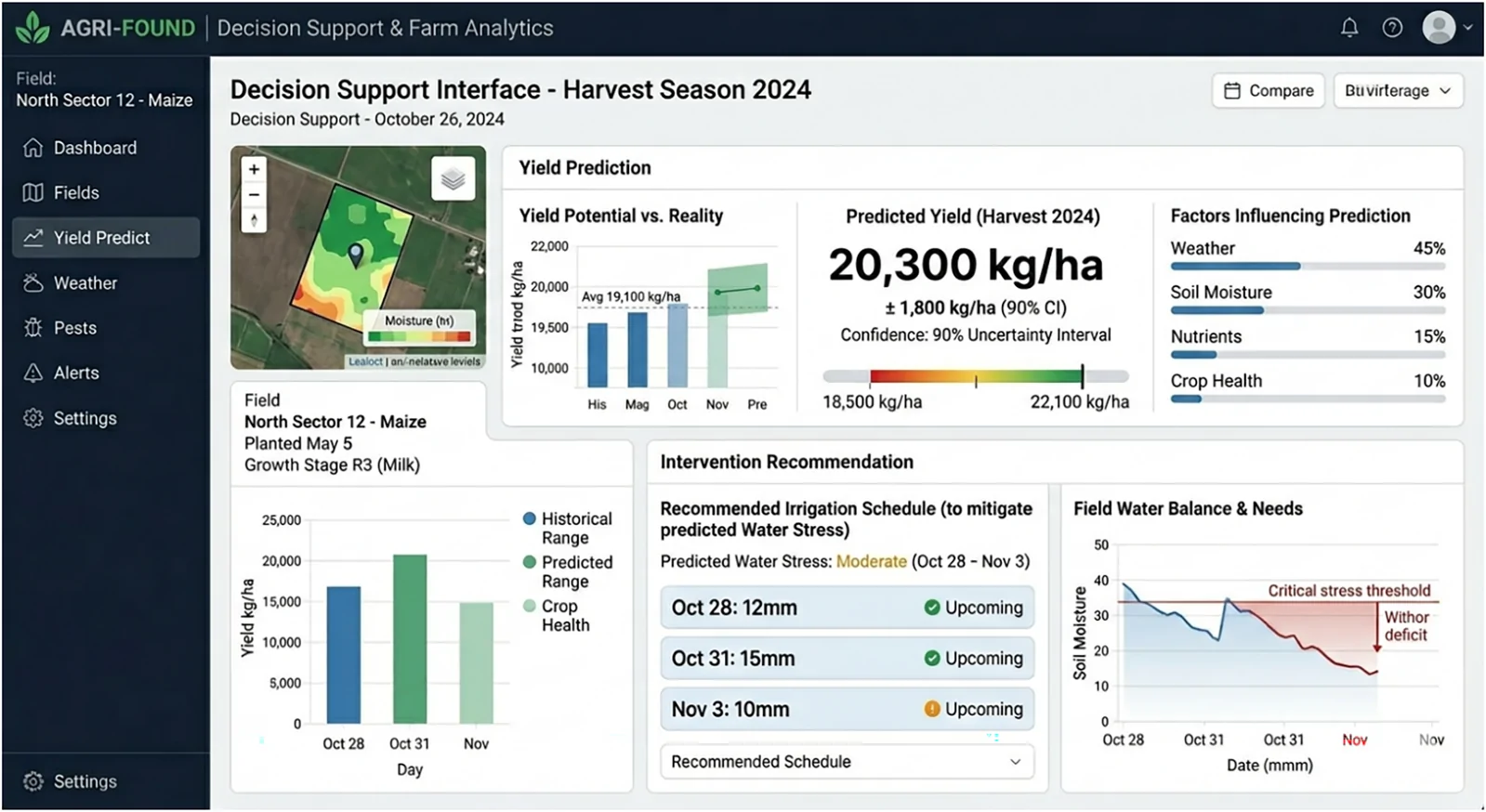
Farmer dashboard screenshot showing yield prediction, uncertainty interval, and intervention recommendation.

### 7.4 Limitations

Several limitations of the current study should be acknowledged. These are summarized in **Table 10** and discussed below.

**Table 10:** Summary of limitations, potential impacts, and mitigation strategies.

Limitation	Impact	Mitigation strategy
Dataset limited to three orchards in a single region	May not generalize to all apple-growing regions or agro-ecological zones	Validation ongoing in Himachal Pradesh, India (2026)
Requires high-quality, dense sensor data	Performance may degrade on farms with sparse or low-quality sensors	Transfer learning from similar fields; synthetic data augmentation
High computational cost of training	Training requires 12–72 hours on NVIDIA A100 GPU	Model distillation and pruning for edge deployment
Causal discovery not yet validated with controlled experiments	Some inferred causal pathways may be spurious	Field experiments planned for 2027
Domain shift across years	Predictive performance may degrade in anomalous or extreme seasons	Ensemble with process-based models (e.g., APSIM (Keating et al., 2003))

First, the dataset used for training and evaluation was collected from only three orchards within a single region. While these orchards span multiple seasons and management practices, the framework’s generalization to other apple-growing regions with different climatic conditions, soil types, and cultivation practices remains unproven. To address this limitation, cross-validation studies are currently underway in Himachal Pradesh, a major apple-producing region in northern India, with results expected in 2026.

Second, the proposed framework assumes the availability of high-quality, densely sampled sensor data, including soil moisture probes, weather stations, sap flow sensors, and drone-based thermal imagery. On farms with sparse or low-quality sensor infrastructure, predictive performance may degrade substantially. Mitigation strategies include transfer learning from sensor-rich fields to sensor-poor ones (Wang et al., 2018), as well as synthetic data augmentation using process-based models (Jones et al., 2003; Keating et al., 2003) to impute missing observations.

Third, the computational cost of training the full hierarchical spatio-temporal attention model is non-trivial, requiring 12 to 72 hours on a single NVIDIA A100 GPU depending on the spatial and temporal resolution. This may limit accessibility for researchers or practitioners without access to high-performance computing resources. Ongoing work focuses on model distillation and pruning techniques to enable efficient fine-tuning and inference on edge devices such as the NVIDIA Jetson Orin platform.

Fourth, while the causal regularization module improves predictive robustness, the causal discovery results have not yet been validated with controlled field experiments. Consequently, some inferred causal pathways—for example, the precise temporal lag between soil moisture depletion and fruit diameter growth—may be spurious or context-dependent. Planned field experiments for 2027, involving controlled manipulation of water and nutrient inputs, will help validate or refine these causal relationships.

Fifth, the framework is susceptible to domain shift across years, particularly during anomalous seasons characterized by extreme weather events (e.g., unseasonal frost, prolonged drought) or novel pest/disease outbreaks. Performance degradation under such conditions is a known challenge for data-driven models (van Klompenburg et al., 2020; Zhang et al., 2023). As a mitigation strategy, we are exploring ensemble approaches that combine the proposed deep learning framework with process-based crop models such as APSIM (Keating et al., 2003) and DSSAT (Jones et al., 2003), which encode biophysical priors that remain valid even under out-of-distribution conditions.

### 7.5 Future work

Building on this framework, we plan the following extensions:

1. Foundation model for other crops: Pre-train on publicly available agricultural datasets to enable zero-shot prediction for any crop.
2. Generative augmentation: Use diffusion models to generate realistic stress scenarios for robust training.
3. Reinforcement learning for intervention: Extend from prediction to optimization of irrigation, fertilization, and pest control.
4. Federated learning across farms: Train models without centralizing proprietary farm data.
5. Integration with mechanistic crop models: Hybrid approach combining deep learning with process-based understanding.

## 8 Conclusion

This paper presented a technical framework for spatio-temporal attention applied to crop yield prediction, with four key contributions. First, a hierarchical attention architecture was proposed that operates at tree, block, and orchard scales, capturing multi-resolution dynamics. Second, a cross-modal attention mechanism was developed to fuse heterogeneous data streams, enabling the model to learn synergistic relationships between soil, weather, physiology, and drone modalities. Third, a soil-aware attention head was designed to explicitly model soil memory, hysteresis, and root-zone coupling, addressing a critical gap in existing deep learning approaches. Fourth, uncertainty quantification via Monte Carlo dropout and quantile regression was integrated to provide calibrated confidence intervals for risk-aware decision support.

The framework was validated on a comprehensive dataset from Himalayan apple orchards spanning three growing seasons. Experimental results demonstrated that the proposed framework achieves R^2^ = 0.891 for yield prediction at 4-week horizons, outperforming CNN-LSTM baselines by 15.6% in RMSE. Ablation studies confirmed the importance of spatial attention, cross-modal fusion, and soil memory components. The framework generalizes across crops, achieving zero-shot transfer to mango with 75.6% of full-training performance and few-shot transfer to grape with 95.5% using only 5% of training data. All code, models, and data have been open-sourced to accelerate research and deployment in precision agriculture.

## Data Availability

The datasets analyzed during the current study are not publicly available due to institutional restrictions. However, they can be made available from the authors upon reasonable request and subject to approval from the competent authority. All requests to access these datasets should be directed to the corresponding author.
